# Mission Hospitals of the Empire: Their Achievement and Position To-Day

**Published:** 1913-11-29

**Authors:** 


### MISSION HOSPITALS OF THE EMPIRE.

### Their Achievement and Position To-day.

In every civilised country of the world in which hospitals have been established medical missionaries have been the pioneers. And even in India, while the Medical Service of the Government has long occupied the more important centres of population, and is gradually extending its beneficent activities even to rural districts, mission hospitals have led the way in several departments of hospital work and in the wilder regions.

Practically the whole existing medical work in China and hospitals for women in India are the most conspicuous proofs of these assertions. There are at the present moment about 800 medical missionaries at work in China. In fact, excepting the treaty ports and a few other places where military surgeons or doctors in charge of the various legations practise, virtually all scientific medical and surgical work in China is in the hands of missionaries. The enlightened statesmen who are now at the head of affairs in China are most desirous to secure for their country the advantages of Western medical science. And, looking round for possible instructors of the rising generation of Chinese doctors, the presence of this large body containing many men with the highest European qualifications has attracted their attention. The desire to make use of them has been an important factor in the changed attitude of the Chinese Government towards Christianity and missions in general.

### Medical Missionaries in China.

Its present policy was shown in a speech reported in the *Times* of January 17, 1913. The China Medical Missionary Association had just held its triennial conference in Peking. Its members, seventy-three in number, were received by the President of the Republic, Yuan Shih-kai, who, in replying to an address presented by the missionaries, paid a warm tribute to them and to their work. He said: "I am delighted to receive the medical missionaries. We are most grateful to you for your charitable services, especially in the interior, where the importance of sanitary principles, once comparatively unknown, is being increasingly recognised owing to your labours. Many of you assisted during the plague, materially aiding in restricting the ravages of the disease which had alarmed the world, while during the revolution many of you faced dangers and difficulties in order to relieve the sufferers. I am glad of this opportunity to offer you our most sincere personal thanks, and I hope that you will continue your labours, thus adding to the glory of your reputation and strengthening the bonds of friendship between your countries and ours." Formerly foreign doctors were looked upon with suspicion in China; dark tales were told of eyes taken out from helpless patients to make medicine, and of patients being killed to obtain certain parts of their bodies, and it was difficult to persuade a native to come into the hospital at all. To-day the hospitals are filled to overflowing, and patients will pay what to them are large sums for the privilege of being treated by the foreign doctor; while to be taken on as a hospital assistant and so get his foot on the ladder which may lead ultimately to a training in Western medicine is the ambition of thousands of the promising youth of China.

### Need for Medical Schools.

One of the greatest needs of China to-day is medical education, and this the missionaries are seeking to supply. In most of the mission hospitals some sort of medical training has been attempted, but what is wanted is a well-equipped and well-staffed medical school in each of the great districts of China. Such a school has been in existence at Peking since 1906 and medical education has been long given at Hangchow, Fuhkien, and other hospitals which are now to be raised to the status of regular medical schools.

In India, where the customs of the country prevent Mahomedan women or those belonging to the respectable Hindu castes from entering a hospital to which men are admitted either as patients or doctors, the mission hospitals were for long the only ones available for women. The Dufferin Hospitals which have been opened under Government auspices were admittedly the outcome of missionary example, though conducted strictly on secular lines and so able to secure the support of wealthy natives. Of the Zenana missionary hospitals the best known is that carried on by the S.P.G. and Cambridge Mission in Delhi. Its work has won it recognition from the Government of India in various ways, and its reputation secured it special honour from her Majesty the Queen during her visit to Delhi for the Durbar in 1911. It is the successor of the first hospital for women and children established in India, but the site has been twice removed and is now just outside the city walls. The work is carried on by four European women doctors, three nursing sisters, and twenty-six Indian nurses and probationers. In 1912 over 1,000 operations were performed.

Similar hospitals have been established by the S.P.G. at Cawnpur and Karnal, and by the Bible and Zenana Medical Mission at Lucknow, Benares, and Patna; while many others in less famous cities are carried on by these and other missionary societies.

The Cawnpur Hospital has a special interest attaching to it from its proximity to the scene of the massacres and has been called the revenge of the women of England for the massacres of the Mutiny, having been erected entirely by subscriptions from English women. The death of several members of the staff from plague caught from their patients had the curious effect of quieting the populace when violent riots had taken place owing to the prevalent idea that the plague had been introduced by the English to diminish the native population.

On the North-West Frontier of India there is a very remarkable chain of mission hospitals, worked by the Church Missionary and Church of England Zenana Missionary Societies. These are attended by many patients from places far outside British territory, and are recognised as having a most pacific effect on the frontier, besides relieving an immense amount of suffering untouched by any other medical agency. In Kashmir, too, the work of the Mission Hospital with its 245 beds is widely famous.

### Native Staffs.

The work of training native nurses, which is carried on at the hospitals at Delhi, Cawnpur, and other places, is peculiarly valuable in view of the widespread ignorance in India of even the most rudimentary ideas of the proper treatment of the commonest diseases, and of women in childbirth. A medical school for women has also been established at Ludhiana in the Punjab, where a better class of women can go and will enter the Government schools to be taught by and with men.

In South India the largest mission hospitals are in the Native States, where the same provision as the Indian Medical Service makes for the needs of the people in British territory does not exist.

From the time of Livingstone Africa has had a special attraction for medical missionaries, and hospitals have been established in a very large number of Anglican and Protestant mission stations in all parts of the Continent. The most remarkable are those of the Church Missionary Society in Uganda and Egypt. The hospital at Mengo in Uganda has 168 beds, and in 1912 the in-patients totalled 2,078. At old Cairo, though the regular hospital beds only number 89 and the in-patients 1,334, a species of resident out-patient accommodation has been established especially for the treatment of that scourge of Egypt, ankylostomiasis, where 300 patients can be housed and 4,812 cases were treated during the year 1912 with remarkable success.

Perhaps nothing has had so great an effect in breaking down the tyranny and power of the witch-doctor in Africa, with all the resultant evils of tribal vengeance and murders, as the success of proper medical and surgical treatment. It has proved a civilising agent of the first importance, and the missions have almost invariably been the pioneers in this. Such was the case in Madagascar, but the French, in pursuance of their anti-religious policy, have now suppressed the work of mission hospitals there, and made provision to some extent for the needs of the people by establishing Government medical aid. Japan affords a somewhat parallel case, as the Government now prefer to provide on a secular basis for the needs which at first had no provision to meet them, except that afforded by mission hospitals.

In Persia there is a remarkable hospital of the Church Missionary Society at Ispahan, with 180 beds and a record of just 600 major operations in 1912. As might be expected from the state of the country, serious gunshot wounds form a large proportion of the cases in Persia, and the mission doctors are called upon to treat Government servants, soldiers, thieves, and highwaymen alike.

Separate mention should be made of leper hospitals, which are carried on by various missions, sometimes in connection with and sometimes quite distinct from other medical work. In uncivilised countries this is a sphere left exclusively to missionaries. The largest leper asylum and hospital in India is at Purulia in Chota Nagpur, carried on by a German mission, but supported by the Mission to Lepers Society of this country. No other medical work is connected with it. This society has many other leper hospitals, both in India and China. Some mission hospitals in those countries set apart a certain number of beds for lepers.

### Mission Hospital Dispensaries.

In studying the statistics available it will be seen that the dispensaries attached to mission hospitals return very large numbers of out-patients, but it is difficult to institute any comparison of numbers between them or with other hospitals—*e.g.*, those under the English Government in India—as a uniform system of statistics of out-patients does not obtain. Some return figures representing "new cases" which should be multiplied by two or three in comparing them with those which return only attendances, where the same patient may be included many times.

But, however computed, the combined numbers show an enormous amount of philanthropic effort at a comparatively trifling cost. The expense of a bed for a year in some of these mission hospitals is estimated as low as £3, in few does it exceed £10, while the average expense of a bed in a London hospital works out at £81. Making allowance for the difference in the cost of living in uncivilised countries from that in England, this indicates a careful management of the funds entrusted to the societies by the benevolent public.

